# Retracted: Immunization Coverage: Role of Sociodemographic Variables

**DOI:** 10.1155/2014/890248

**Published:** 2014-12-29

**Authors:** Advances in Preventive Medicine

The paper titled “Immunization Coverage: Role of Sociodemographic Variables” [1], published in Advances in Preventive Medicine, has been retracted upon the authors' request due to a flaw in data acquisition.
